# Autophagy Is Involved in the Reduction of Myelinating Schwann Cell Cytoplasm during Myelin Maturation of the Peripheral Nerve

**DOI:** 10.1371/journal.pone.0116624

**Published:** 2015-01-12

**Authors:** So Young Jang, Yoon Kyung Shin, So Young Park, Joo Youn Park, Seo-Hee Rha, Jong Kuk Kim, Hye Jeong Lee, Hwan Tae Park

**Affiliations:** 1 Department of Physiology, Mitochondria Hub Regulation Center, Busan, Korea; 2 Department of Pharmacology, Mitochondria Hub Regulation Center, Busan, Korea; 3 Department of Pathology, College of Medicine, Dong-A University, Busan, Korea; 4 Department of Neurology, College of Medicine, Dong-A University, Busan, Korea; Toho University School of Medicine, JAPAN

## Abstract

Peripheral nerve myelination involves dynamic changes in Schwann cell morphology and membrane structure. Recent studies have demonstrated that autophagy regulates organelle biogenesis and plasma membrane dynamics. In the present study, we investigated the role of autophagy in the development and differentiation of myelinating Schwann cells during sciatic nerve myelination. Electron microscopy and biochemical assays have shown that Schwann cells remove excess cytoplasmic organelles during myelination through macroautophagy. Inhibition of autophagy via Schwann cell-specific removal of ATG7, an essential molecule for macroautophagy, using a conditional knockout strategy, resulted in abnormally enlarged abaxonal cytoplasm in myelinating Schwann cells that contained a large number of ribosomes and an atypically expanded endoplasmic reticulum. Small fiber hypermyelination and minor anomalous peripheral nerve functions are observed in this mutant. Rapamycin-induced suppression of mTOR activity during the early postnatal period enhanced not only autophagy but also developmental reduction of myelinating Schwann cells cytoplasm *in vivo*. Together, our findings suggest that autophagy is a regulatory mechanism of Schwann cells structural plasticity during myelination.

## Introduction

The myelination of peripheral nerves is achieved by wrapping of the plasma membranes of myelinating Schwann cells (mSCs) around the axons during postnatal development [1]. Myelination appears to require a continuous change of SC cytoplasm, because the volumes of the axon and the number of myelin lamellae grow until the maturation of myelination is complete. When promyelinating SCs begin to myelinate axons, SCs have large cytoplasm containing numerous mitochondria, ribosome and endoplasmic reticulum [2–4]. As the number of lamellae increased, cytoplasm between lamellae is excluded and confined to external mesaxon and abaxonal cytoplasm. Abaxonal SC cytoplasm is abundant until the maximum rate of myelin addition reaches around the end of second postnatal weeks in rodents [2]. Slow-down of the accumulation of myelin lamellae is then accompanied by the reduction of abaxonal cytoplasmic volume, resulting in little cytoplasm and few organelles in the abaxonal areas outside of the compact myelin in adult mSCs [2, 3]. It is still unknown whether an active cytoplasmic degradation mechanism including autophagy regulates cytoplasmic exclusion during myelin compaction and the substantial reduction of abaxonal cytoplasmic volume in the maturation period. Furthermore, the possibility of these cytoplasmic changes influencing the functional parameters of SC differentiation, such as the extent of myelination and longitudinal growth, has not been determined yet.

Macroautophagy is the nonselective lysosomal degradation of cytosolic organelles and proteins [5]. During macroautophagy, cell organelles and cytosol are sequestered into autophagosomes that are derived from the endoplasmic reticulum or other membrane sources, and lysosomal fusion with autophagosomes is the final step in this degradation process. The recent discovery of the molecular mechanisms of macroautophagy identified numerous autophagy-related proteins (ATGs) [6]. ATG complexes participate in the stepwise reactions resulting in autophagosome formation, and studies of mice with tissue-specific knockouts of critical autophagy genes such as *atg7* have revealed that autophagy is important for several physiological processes, including the differentiation of neuronal and non-neuronal tissues [7–10]. In the present study, to determine the function of autophagy in the morphological changes of SC cytoplasm as well as myelination, we generated SC-specific *atg7* conditional knockout mice. Selective loss of the *atg7* gene in SCs resulted in the accumulation of excess cytosol and organelles in the abaxonal areas of mature mSCs cells and minor changes in small fiber myelination. In addition, we found that macroautophagy is developmentally regulated in the peripheral nerves during postnatal life. Our results indicate that autophagy is a regulatory mechanism of SC structural plasticity during myelination.

## Materials and Methods

### Materials

Antibodies against beta-actin, LAMP1, P0, p62 and p75NTR were purchased from Santa Cruz Biotechnology (Santa Cruz, CA). Anti-phospho-ERK, anti-phospho-AKT and anti-phsopho-p70 S6 kinase antibodies were obtained from Cell Signaling Lab (Beverly, MA). Antibodies against MBP and S100 were obtained from Abcam and Chemicon, respectively. Antibodies against ATG7 were obtained from Sigma (St. Louis, MO) and Cell Signaling Lab. Antibody against LC3B was purchased from Invitrogen (Carlsbad, CA). All other undesignated reagents were purchased from Sigma.

### Generation and characterization of Schwann cell-specific atg7 knockout mice

The atg7flox/flox mice (atg7^*flox*^ obtained from Dr. Masaaki Komatsu [11]) and P0-Cre transgenic mice (obtained from Dr. Wrabetz [12]) were crossed to produce the SC-specific atg7 conditional knockout mice (atg7-SCKO). Atg7^*flox*^ mice were used as controls in all experiments. The genotypes of the mice were determined with PCR using following primers: Forward P0-Cre: CCACCACCTCTCCATTGCAC, Reverse P0-Cre:GCTGGCCCAAATGTTGCTGG, Forward ATG7: TGGCTGCTACTTCTGCAATGATGT, Reverse ATG7:CAGGACAGAGACCATCAGCTCCAC. The recombination resulted in downregulation of ATG7 mRNA in the sciatic nerves, and mRNA levels were analyzed by qPCR with following primers for ATG7; Forward:CCAAAAGGCTGGCTGAGTCA, Reverse: CCAATCGCCAGCACATCA.

### Electron microscopic (EM) analysis and morphometry

Sciatic nerves were processed for electron microscopy as described previously [13]. All surgical procedures were performed according to protocols approved by the Dong-A University Committee on animal research (Permit number: DIACUC-13–11), which follows the guidelines for animal experiments that were established by the Korean Academy of Medical Sciences. After anesthesia with high doses of ketamine hydrochloride (Sanofi-Ceva, Düsseldorf, Germany) and Rompun (Bayer, Leverkusen, Germany), sciatic nerves were removed and immersed in a fixative (2.5% glutaraldehyde and 2.0% paraformaldehyde [PFA] in 0.1 M sodium phosphate buffer, pH 7.4) at 4°C overnight. Nerves were post-fixed in 1% osmium tetroxide in the same buffer for 2 h and embedded in Epon after alcohol dehydration. Semithin sections were stained with toluidine blue, and ultrathin sections were stained with 5% uranyl acetate and Reynold’s lead citrate. Sections were examined on a Hitach electron microscope equipped with a digital camera. For morphometric analysis, at least three independent experiments were performed.

**Figure 1:**
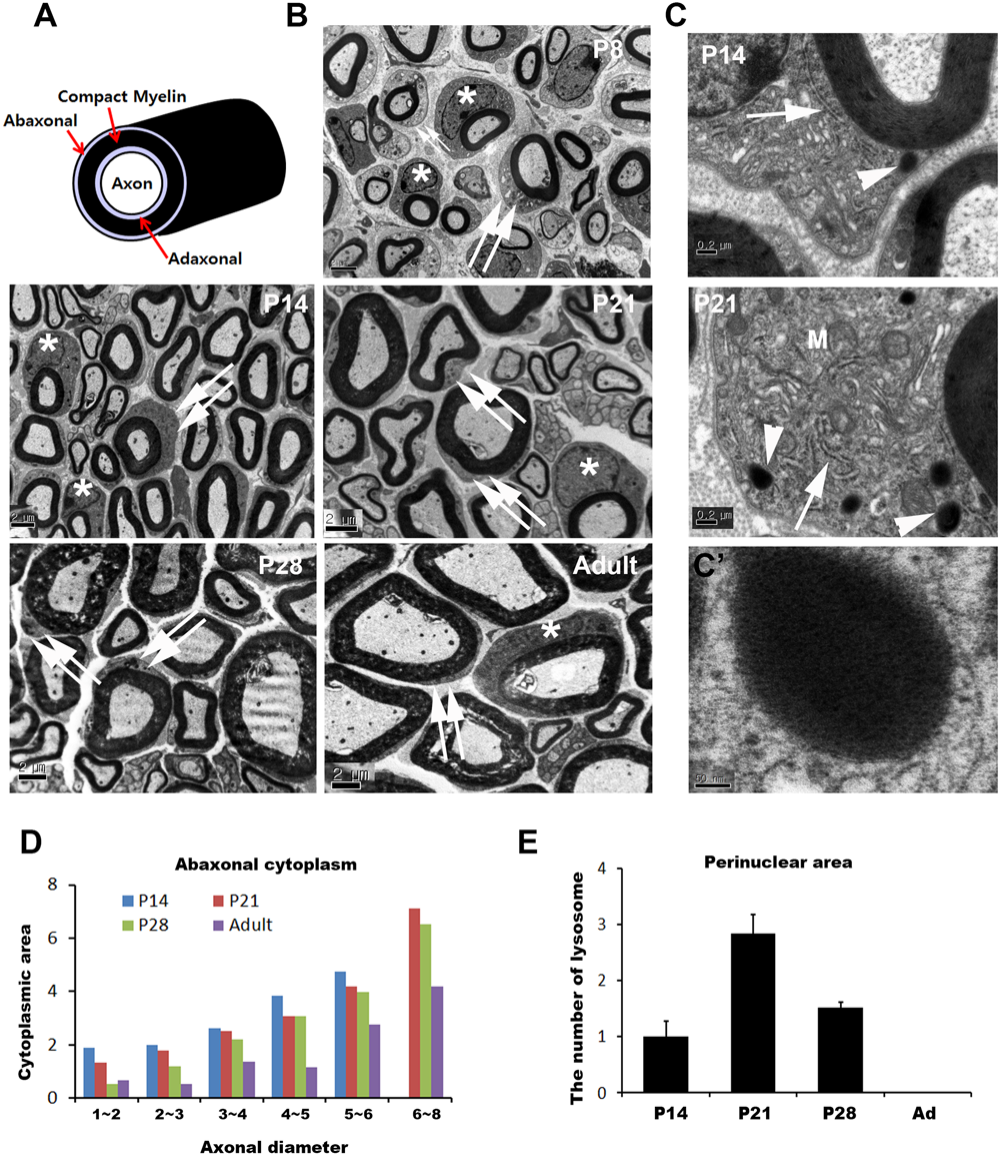
Changes of cytoplasmic area of mSCs during the postnatal period. A. A schematic drawing of mSCs showing the abaxonal cytoplasm outside of the compact myelin sheath. B. Representative EM images of sciatic nerves from wild-type mice showing the reduction of cytoplasmic area of mSCs during postnatal development. Asterisk; nucleus, double arrows; abaxonal cytoplasm. Note the developmental downregulation of abaxonal cytoplasm. C. Representative EM images of mSCs from mice of different ages. Arrowheads: lysosome. M: mitochondria. N: nucleus. Arrows demonstrate various morphologies of RER. C’. Enlarged image of a lysosome. D. Morphometric quantification of the abaxonal cytoplasmic area (n = 3 for each ages). E. The number of lysosomes in the perinuclear cytoplasm of mSCs from different ages was counted under EM, and the mean number of lysosomes per mSC is displayed (n = 3, mean±SEM).

For electron microscopic analysis, captured EM images (×4000~5000) were analyzed using the ImageJ software (National Institute of Health, Bethesda). Abaxonal areas were demarcated and calculated by drawing a continuous line with the ImageJ software and mean areas were calculated from approximately 300~500 randomly selected myelinated SCs at each time point (n = 3). The number of lysosomes in the perinuclear cytoplasm of 70~100 mSCs that had nuclei visible in ultrathin sections was counted, using three animals in each age group. Lysosome was defined as a membrane-bound electron dense structure (0.2~0.5 μM in diameter, Fig. 1C’) which was distinguished from membrane-unbound less electron-dense lipid droplets. Quantitative analysis of the number of appositions in the mSCs was performed from 300~500 randomly selected myelinated SCs at P60 (n = 3). G ratios were determined by dividing the perimeter of the axon by that of the outset leaflet of the myelin sheath at P10 and P60. Approximately 170~200 round axons from two mice in each group were employed for the analysis. The morphometric analysis of Remak bundle development (the number of axons in a bundle) was determined from the same images used for g ratio analysis and approximately 130 Remak bundles from two animals (P60) in each group were pooled and analyzed.

The number of myelinated fibers was counted with a montage that consisted of approximately 20 photographs taken at ×600 magnification using semithin sections (n = 3). The mean length of the internodes was measured using 130 teased nerve fibers in each group at P21 (n = 3).

### Sciatic nerve processing for immunofluorescence (IF) staining

At postnatal days (P) 8, 14, 21, 28 and adulthood (P60), animals were sacrificed with high doses of ketamine hydrochloride and Rompun. The sciatic nerves were removed and fixed in 4% PFA for 1 day, and the nerves were cryoprotected in a 20% sucrose solution. Cross sections with 14 μm thickness were made using a cryostat (Frigocut, Leica), and the section were stored in a deep freezer until use. For the teased nerve preparations, fixed nerves were teased and mounted on a slide under a stereomicroscope. The slides were blocked with phosphate buffered saline (PBS) containing 0.2% Triton X-100 and 2% bovine serum albumin for 1 h. The sections were then incubated with primary antibodies against LC3B (1;1000), LAMP1 (1;500), S100 (1;1000) and MBP (1;2000) for 16 h at 4°C and washed three times with PBS. Next, the slides were incubated with an Alexa 488 or Texas-red conjugated secondary antibody for 3 h at room temperature. The sections were then washed three times with PBS, and coverslips were adhered to glass slides with a mounting medium (Gel Mount, BioMeda, USA) and viewed under a laser confocal microscope (LSM510, Carl Zeiss, Germany). To obtain quantitative analysis of the staining, the intensity of the immunofluorescent staining was measured from two randomly selected 272×272 µm areas from one section using the Zen 2009 software, and a total of 8~10 sections from 3 independent experiments for each group were used for capturing images with a 20×/0.50 Plan-Neofluar lens (Carl Zeiss). The intensity of the fluorescence was categorized into 5 grades ranging between 0–250 (i.e., an intensity unit between 0–50 was the lowest intensity grade whereas a unit between 200–250 considered the highest grade), and the relative intensity of the staining was demonstrated by counting the number of the pixels with an intensity unit over 50, ranging from 0–250 units.

### Western blot analysis

For Western blot analysis, sciatic nerves were harvested and homogenized with a Polytron homogenizer in a modified radioimmune-precipitation assay buffer (150 mM NaCl, 1% NP-40, 1 mM EDTA, 0.5% deoxycholic acid, 2 µg/mL aprotinin, 1 mM PMSF, 5 mM benzamidine, 1 mM sodium orthovanadate, and 1×protease inhibitor cocktail [Roche, Indianapolis, IN]). The lysates were centrifuged at 8,000g for 10 min at 4°C, and the supernatant was collected. Protein (25~35 μg) was separated by SDS-PAGE, and then transferred onto a nitrocellulose membrane (Amersham Biosciences). After blocking with 0.1% Tween-20 and 5% nonfat dry milk in Tris buffered saline (pH. 7.2, TBS) at room temperature for 1 h, the membranes were incubated with primary antibodies (1:500–1,000) in TBST containing 2% nonfat dry milk at 4°C overnight. After three 15 min washes with TBST, the membranes were incubated with a horseradish peroxidase-conjugated secondary antibody (1:3,000) for 1 h at RT. The signals were detected using the ECL system (ECL Advance kit, Amersham Biosciences). For quantitative analysis, the films from 3 independent experiments (n = 3) were scanned and the intensity was analyzed using a LAS image analysis system (Fujifilm, Japan).

### Rapamycin injection

Rapamycin (Cell signaling Labs) was dissolved in a vehicle solution containing 5% polyethyleneglycol 400, 5% Tween 80 and 4% ethyl alcohol. Rapamycin or vehicle was administered daily via intraperitoneal injection at 5 mg/kg for 7 days beginning at P7. One day after the final injection (P14), the animals were sacrificed and the sciatic nerves were processed for EM and Western blot analysis (n = 3).

### Electrophysiology

Electrophysiology was performed on sciatic nerves of mice (n = 4 for each genotype) at 60~70 days of age. After anesthesia, a pair of steel needle electrodes (Technomed Europe, Netherland) was placed over sciatic nerves at sciatic notch for proximal stimulation. A second pair of electrodes was placed above the ankle for distal stimulation. Motor nerve conduction study was performed with supramaximal stimulations of 0.01ms duration using a Nicolet Viking IV (Madison, WI, USA). The peak to peak amplitude and latency of CMAP was recorded from foot muscles using steel electrodes. In addition, we measured F-wave latency that was generated by backfiring of antidromically activated motoneurons. The distance between the two sites of stimulation was measured alongside the skin surface with fully extended legs and nerve conduction velocity was calculated by dividing the latency with the distance.

### Statistical analysis

Statistical analysis was performed by using GraphPad Prism software (GraphPad, San Diego, CA, USA). P values are from Student’s two-tailed test, and results were expressed as mean and SEM.

## Results

### Autophagic reduction of myelinating Schwann cell cytoplasm during postnatal development

We examined the temporal changes in cytoplasmic area of mSCs during postnatal development by measuring the abaxonal cytoplasmic areas around the compact myelin sheath using EM. Promyelinating SCs and active mSCs in the early postnatal period (P8, P14) had large cytoplasmic areas in both the perinuclear and the abaxonal cytoplasm (Fig. 1B, C). However, mature mSCs at adults typically contained abundant cytoplasm only around the perinuclear areas, but the abaxonal cytoplasmic areas outside the compact myelin were very thin with few organelles (Fig. 1A, B). Indeed, measurement of the abaxonal areas revealed a developmental downregulation of abaxonal cytoplasmic area after P14 (Fig. 1D), suggesting the occurrence of cytoplasm reduction during myelin maturation.

At postnatal day 14 (P14)-P28, the abaxonal and perinuclear cytoplasm of the mSCs displayed an extensive network of membranous organelles, such as rough endoplasmic reticulum (RER), Golgi complexes and endosomes, that sometimes appeared to make autophagosomes (Fig. 1C). In contrast to the mSCs in adults, electron dense lysosomes or autolysosomes were also frequently found near the membranous network in the cytoplasm of mSCs at P14–28 (Fig. 1C, E). We counted the number of lysosomes in the perinuclear area of mSCs from different ages and found that the number was greatest at P21, whereas no lysosomes were observed in the mSCs of adults (Fig. 1E). These EM findings suggest that macroautophagy may participate in the reduction of mSC cytoplasm during the postnatal myelination period.

### The canonical autophagic pathway is activated in Schwann cells during postnatal development

**Figure 2:**
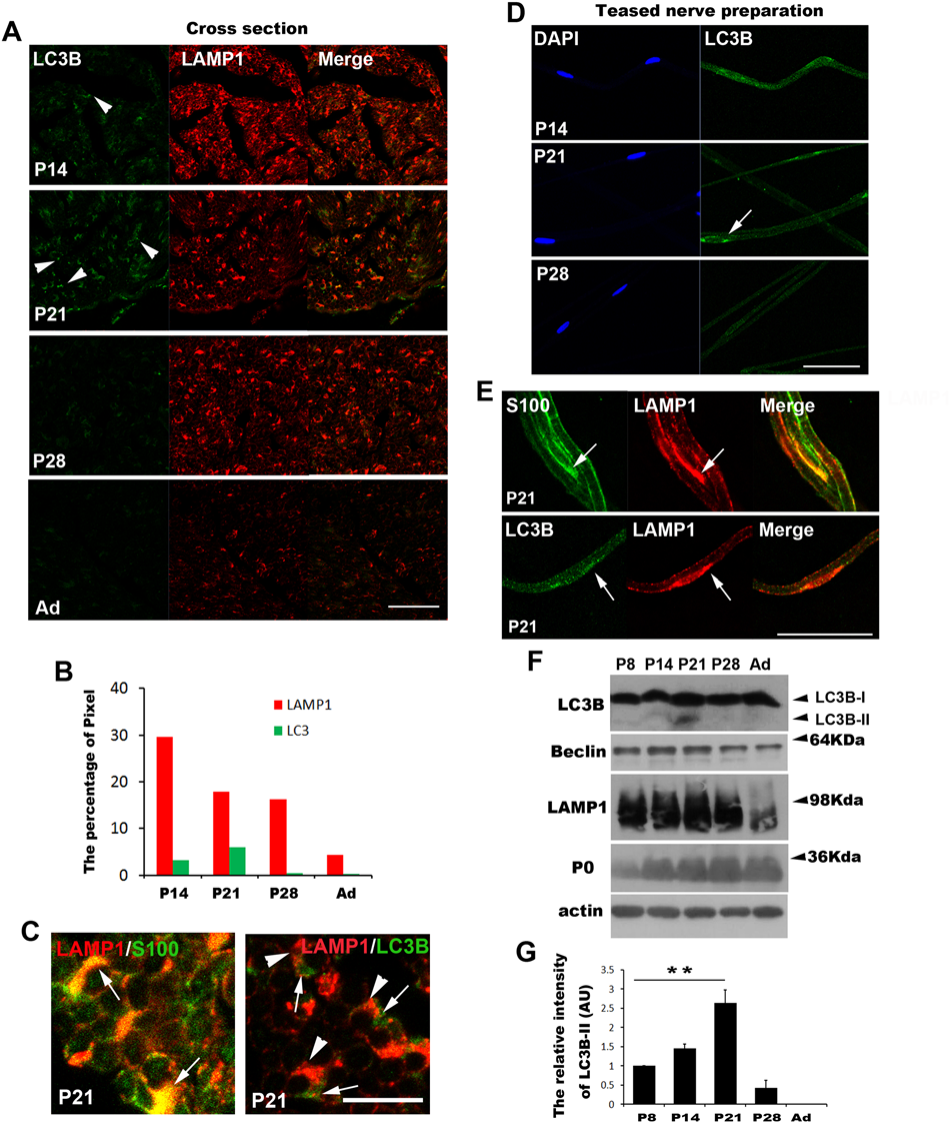
Canonical autophagy is activated in the sciatic nerves during postnatal development. A-C. Representative IF micrographs from transverse sciatic nerve sections showing the dynamic patterns of LC3B expression and puncta formation (arrowheads) as well as LAMP1 expression during postnatal development (P14~P60 [Ad]). Note the peak LC3B immunostaining at P21 and subsequent downregulation thereafter. Scale bar = 50 μm. B. For quantitative analysis of LC3B and LAMP1 staining, the relative intensity of the staining was calculated by counting the number of pixels with an intensity unit over 50 from 0–250 units (Materials and Methods). C. Left figure; LAMP1 (red) was co-localized with S100 (green, a SC marker) (arrows) in mSCs. Right figure; LC3B staining (arrow, green) was juxtaposed with LAMP1 staining (arrowheads, red). D, E. Representative IF micrographs from a teased nerve preparation of sciatic nerves showing LC3B (D) and SC expression of LAMP1 (E) (arrow). Scale bar = 50 μm. F, G. Western blot analysis of sciatic nerve extracts from animals at P8~P60. Note the peak level of the LC3B-II form at P21. G. Quantitative analysis of the LC3B-II levels. The level of LC3B-II at P8 is an arbitrary unit (AU) of 1 (n = 3, mean±SEM). **P<0.01.

To determine whether autophagy occurs in the sciatic nerves during postnatal development *in vivo*, we analyzed the lipid modification of the cytosolic form of microtubule associated protein light chain 3B-I (LC3B-I) to LC3B-II (Fig. 2). The LC3B-II conversion of LC3B-I results in faster migration in SDS-PAGE and LC3B puncta formation in IF staining, which represents canonical ATG-dependent autophagosome formation [14]. IF staining of cross sections of developing sciatic nerves showed diffuse and punctate LC3B staining at P14, and LC3B immunoreactivity was further increased at P21, particularly around the perinuclear area, and then was profoundly decreased at P28 and in adulthood (Fig. 2A–C). Lysosomes were stained with an antibody against lysosomal associated membrane protein 1 (LAMP1). The LAMP1 staining revealed a high level of LAMP1 expression in the sciatic nerves at P14~P28, compared with very low levels of expression in adulthood (Fig. 2A, B, F). At P21, LC3B staining and LAMP1 staining in the cytoplasm of mSCs were located very close to each other, but did not overlap completely (Fig. 2C). The IF staining in a teased nerve preparation also showed similar expression profiles of LC3B and LAMP1 in mSCs, which was identified by S100 staining, with the highest level of staining found in the perinuclear region at P21 (Fig. 2D, E). Western blot analysis with sciatic nerve lysates revealed that LC3B-II levels peaked at P21, and significantly decreased thereafter (Fig. 2F, G). The expression of Beclin-1, a molecule involved in the nucleation of autophagosomal membranes [5], and LAMP1 also showed a postnatal developmental increase until P21, and then was downregulated at P28 and in adults (Fig. 2F). Taken together, these findings indicate that autophagy is activated in mSCs during the postnatal myelination period and then is suppressed in adulthood.

### ATG7 in Schwann cells is essential for reduction of abaxonal cytoplasm during postnatal development

**Figure 3:**
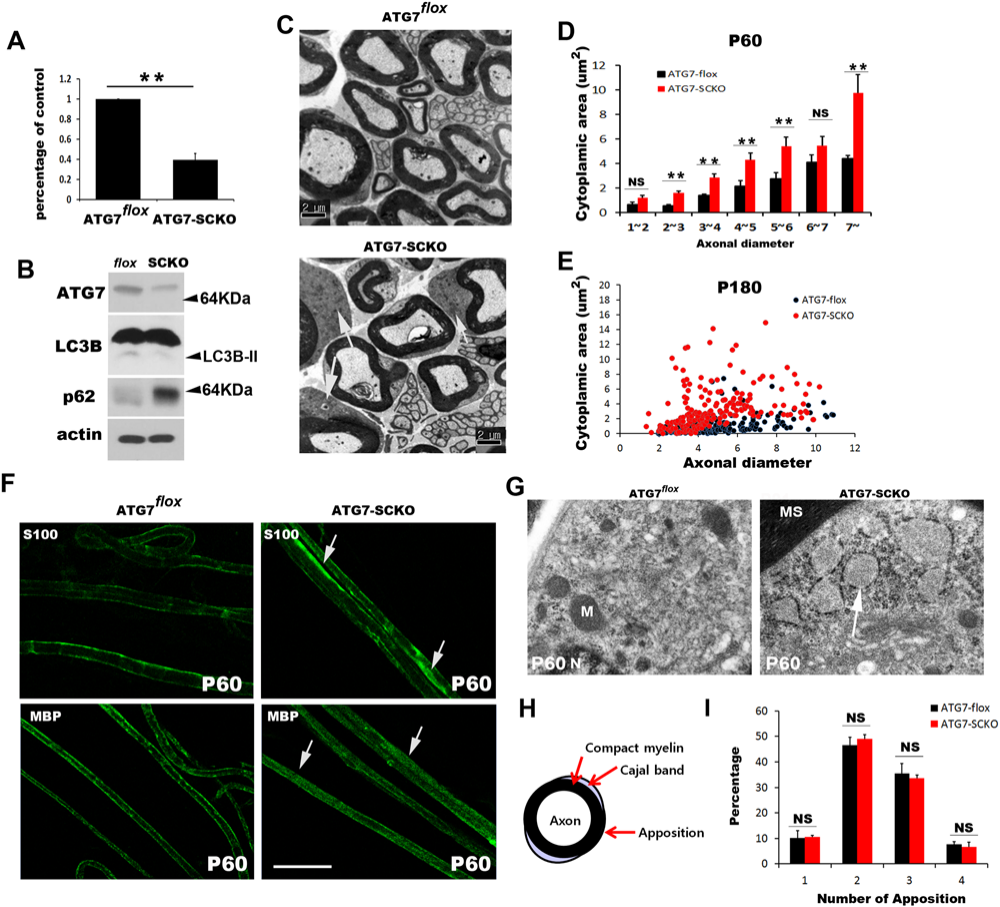
*atg7*-SCKO mice exhibited a failure in the reduction of cytoplasmic area of mSCs. A. qPCR quantification of the relative *atg7* mRNA levels (normalized to β-actin expression) in the sciatic nerves from *atg7^flox^* and *atg7*-SCKO mice in adulthood (n = 3, mean±SEM). ** *P*<0.01. B. Western blot analysis showing ATG7, LC3B-II and p62 levels in *atg7*-SCKO mice, compared to *atg7^flox^* mice at P21. C. Representative EM images of sciatic nerves from *atg7^flox^* and *atg7*-SCKO mice at P60 showing excess residual abaxonal cytoplasm in the mSCs (arrows) in *atg7*-SCKO mice. D. Quantitative analysis of the abaxonal cytoplasmic area at P60 (n = 3, mean±SEM). NS: not significant. **p<0.01. E. Scatter plot showing the cytoplasmic area in relation to the axonal diameter at P180. Approximately 200 axons from two mice in each group were employed for the analysis. F. Representative IF micrographs of a teased nerve preparation showing the abnormal accumulation of S100 and MBP in the cytoplasm of mSCs from *atg7*-SCKO mice (arrows). Scale bar = 50 μm. G. Representative EM images of the mSCs from *atg7*-SCKO mice at P60 showing numerous ribosomes with abnormally expanded RER (arrow) in the residual abaxonal cytoplasm of the mSCs. N; nucleus, MS; myelin sheath, M; mitochondria. H. A schematic drawing showing the Cajal band and membrane apposition. I. Quantitative analysis showing the number of appositions in the mSCs from *atg7^flox^* and *atg7*-SCKO mice at P60 (n = 3, mean±SEM).

To determine the function of autophagy in SCs during peripheral nerve development, we inactivated the ATG7 gene, an autophagy gene encoding an E1-like enzyme essential for autophagosome formation, in a SC-specific manner. We crossed mice homozygous for *atg7* “floxed” (*atg7^flox^*) [11] with mice carrying a P0-Cre transgene [12]. In P0-Cre mice, recombination occurs between E13.5~14.5 specifically in SCs [12]. *Atg7*-SC-specific knockout (SCKO) mice were generated in accordance with Mendelian ratios and did not display overt clinical phenotype in the first 9 months of age. To confirm the specific knock out of endogenous *atg7* in the peripheral nerves, we examined *atg7* mRNA expression in the sciatic nerves of *atg7*-SCKO and *atg7^flox^* control mice using qPCR, and found a significant reduction in *atg7* mRNA in the sciatic nerves of *atg7*-SCKO mice (Fig. 3A). Western blot analysis revealed that ATG7 protein expression was diminished in the sciatic nerves of *atg7*-SCKO mice compared to *atg7^flox^* mice (Fig. 3B). The considerable level of ATG7 staining remaining in the protein lysates of *atg7*-SCKO mice might be due to the ubiquitous expression of ATG7 in axons and fibroblasts. Additionally, we found that a substantial decrease in LC3B-II levels in *atg7*-SCKO mice compared to *atg7^flox^* mice at P21 using Western blot analysis). In addition to this, autophagy deficiency in SCs resulted in the increase of protein levels of p62 in the sciatic nerves (Fig. 3B). Taken together, these findings indicate that autophagy is inactivated in *atg7*-SCKO mice.

We next examined whether a reduction in autophagy during postnatal development would result in abnormal cytoplasmic morphology of mSCs in adulthood (P60 and P180) using EM. In contrast to the very thin abaxonal cytoplasm of mSCs from *atg7^flox^* mice, many mSCs from *atg7*-SCKO mice contained large volume of cytoplasm in abaxonal regions at P60 and P180 (Fig. 3C–E). We measured the abaxonal cytoplasmic areas from mSCs of *atg7^flox^* and *atg7*-SCKO mice, and the analysis indicated a significant increase in cytoplasmic area in the mutant mice (Fig. 3D, E). In the teased nerve preparation, S100 was distributed in a long patch-like pattern in *atg7*-SCKO nerves, which was not observed in *atg7^flox^* mice (Fig. 3F). In addition, the distribution of myelin basic protein (MBP), a myelin protein localized on the cytoplasmic face of the myelin membrane, also showed a diffuse pattern in *atg7*-SCKO nerves (Fig. 3F), suggesting the localization of these proteins in the excess residual cytoplasm of mutant mSCs. The large volume of excess cytoplasm in the mSCs from *atg7*-SCKO mice contained many ribosomes, which were frequently on abnormally enlarged RER (Fig. 3G). These experiments suggest that *atg7*-dependent autophagy is required for the reduction of cytoplasmic area of mSCs during postnatal development.

The cytoplasm outside compact myelin can be divided into a characteristic cytoplasmic channel, the Cajal band, and an apposition where the outer plasma membrane and myelin make contact [15] (Fig. 3H). Because the cytoplasmic areas of mSCs in *atg7*-SCKO mice were abnormal, we investigated the development of this organization of cytoplasmic regions of mSCs by counting the number of membrane appositions. We found no significant difference in the number of appositions between *atg7^flox^* mice and *atg7*-SCKO mice (Fig. 3I), suggesting that the anomalous expansion of abaxonal cytoplasm in autophagy-deficient SCs may not be caused by abnormal development of the cytoplasmic channel.

### Small fiber hypermyelination and abnormal peripheral nerve function in Schwann cell-specific ATG7 knockout mice

**Figure 4:**
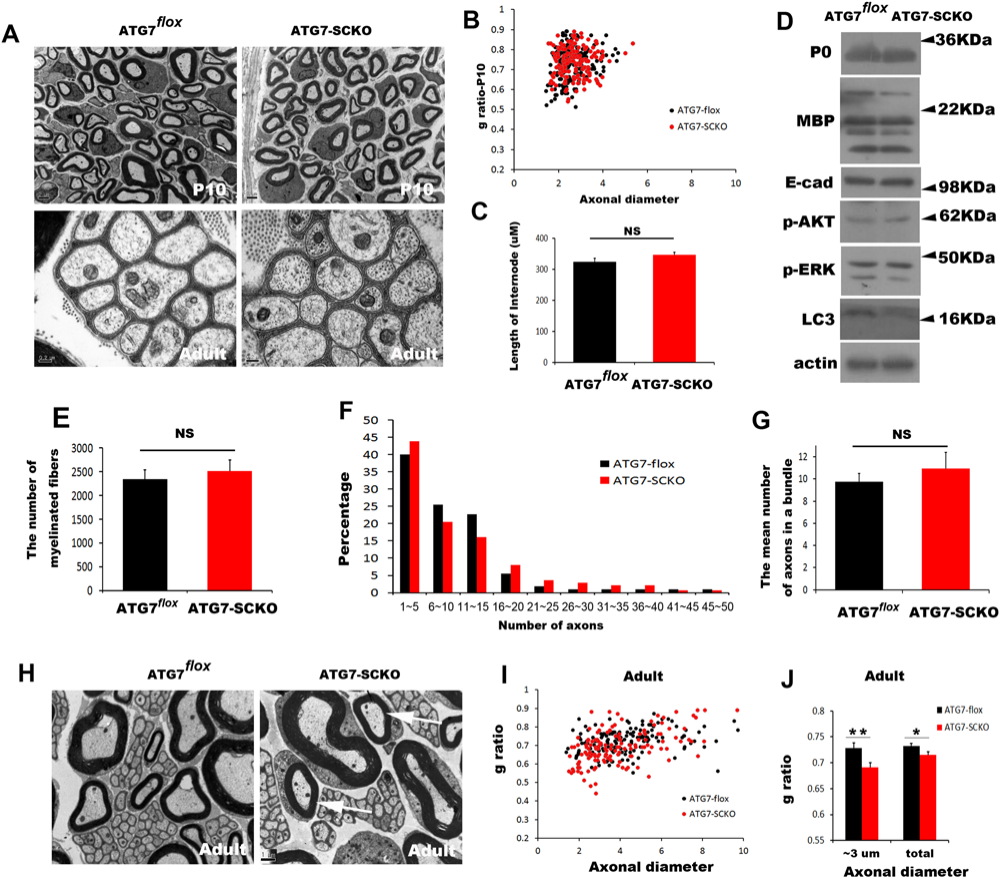
Myelination profiles of sciatic nerves in *atg7*-SCKO mice. A. Representative electron micrographs of sciatic nerve cross sections from animals at P10 and in adulthood. General patterns of myelination at P10 and Remak bundle development were normal in *atg7*-SCKO mice compared with *atg7^flox^* control mice. B. This scatter plot showing the g-ratio in relation to the axonal diameter at P10 displays normal myelination profiles in *atg7*-SCKO mice. C. The mean length of the internodes was measured using 130 teased nerve fibers in each group at P21 (mean±SEM). NS; *p*>0.05. D. Western blot analysis using sciatic nerve extracts from mice at P10. E. The number of myelinated fibers in the sciatic nerves. (n = 3, mean±SEM). NS; *p*>0.05. F. Frequency distribution profile of the number of axons per Remak bundle in *atg7^flox^* control and *atg7*-SCKO mice. G. The mean number of axons in a Remak bundle in the sciatic nerves at P60 (n = 3, mean±SEM). NS; *p*>0.05. H. Representative electron micrographs of sciatic nerve cross sections from adult mice showing hypermyelination of small fibers (arrows). I. Scatter plot showing the g-ratio in relation to the axonal diameter in adulthood. J. Morphometric quantification of the g-ratio of adult mice showed hypermyelination of small fibers that have diameters less than 3 μm. (n = 3, mean±SEM). **P<0.01, *P<0.05.

To determine whether early myelination processes are altered in *atg7*-SCKO mice, we first examined the myelination profiles at P10 using EM and Western blot analysis. Analysis of the g-ratios indicated a normal myelination profile in *atg7*-SCKO mice at P10, compared with *atg7^flox^* mice (Fig. 4A, B). The expression levels of myelin protein zero, MBP and E-cadherin, as well as the phosphorylation levels of ERK and AKT, two important signaling molecules implicated in myelination [1], were not altered in mutant nerves compared with control nerves at P10 (Fig. 4D). In addition, the length of the internodes, which was measured on teased nerve fibers, and the number of myelinated fibers in mutant mice at P21 were not significantly different from that observed in *atg7^flox^* mice (Fig. 4C, E, p>0.05). The morphometric analysis of Remak bundles showed a normal frequency distribution pattern of unmyelinated axons in non-mSCs from *atg7*-SCKO mice at P60 (Fig. 4F). The mean number of axons in a Remak bundle from the mutant mice was not significantly different from that of the control mice (Fig. 4G, p>0.05). Together, these findings suggest that autophagy in SCs might not be involved in axonal sorting and early myelination processes.

We next investigated the myelination profiles of adult sciatic nerves from *atg7*-SCKO mice under ultrastructural observation (Fig. 4H–J). Measurement of the g-ratio showed mild but significant hypermyelination in mutant sciatic nerves (0.7152±0.0056) at P60 compared to that of *atg7^flox^* mice (0.7326±0.005, p<0.05). The distribution of g-ratios indicated that the decrease in the g-ratio was due to an obvious hypermyelination of small axons whose diameters were less than 3 μm (0.7287±0.0097 [*atg7^flox^*] vs 0.691±0.0089 [*atg7*-SCKO], p<0.001) (Fig. 4J). At P180, the hypermyelination phenotypes of small fibers were still observed, but anomalous hypermyelination profiles such as tomacula were not found (data not shown). To determine whether a loss of *atg7* in SCs affects peripheral nerve function, we examined the nerve conduction velocity (NCV) of sciatic nerves at age P60 by measuring the compound muscle action potential (CMAP) (Table 1). Motor NCV and the appearance of proximal F wave not significantly altered in *atg7* mutant mice compared to controls (*p*>0.05). However, we found a significant increase in CMAP amplitude in *atg7*-SCKO mice compared to *atg7^flox^* mice.

**Table 1:** Electrophysiological analysis of *atg7^flox^* control and *atg7*-SCKO mice.

	***atg7^flox^* control mice**	***atg7*-SCKO mice**	***P* value**
Proximal F-wave latency (ms)	5.500±0.315	6.033±0.0093	0.106 (NS)
Distal F-wave latency (ms)	6.840±0.246	6.953±0.0093	0.660 (NS)
NCV	27.56±1.172	25.13±0.576	0.070 (NS)
Proximal amplitude (mv)	2.723±0.281	4.028±0.203	0.001
Distal amplitude (mv)	3.728±0.519	5.029±0.223	0.027

Four animals at age P60~70 in each group were tested.

NS; not significant, Student’s two-tailed test

### Inhibition of the mTOR pathway enhances autophagic reduction of abaxonal cytoplasmic area

**Figure 5:**
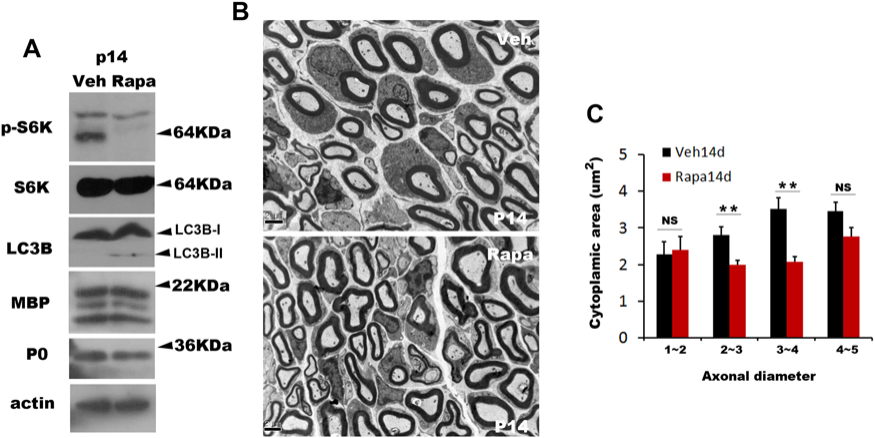
Rapamycin suppressed the mTOR pathway and enhanced cytoplasmic changes. A. Western blot analysis showing the effect of rapamycin injection on the phosphorylation of S6 kinase (p-S6K) and LC3B modification in the sciatic nerves. B. Representative electron micrographs of sciatic nerve sections from animals at P14 after intraperitoneal injection of vehicle (Veh) or rapamycin (Rapa) for one week. C. Quantitative analysis of the abaxonal cytoplasmic area of the mSCs at P14 following injection (n = 3, mean±SEM). NS; not significant. **p<0.01.

The mammalian target of rapamycin (mTOR) pathway is a suppressor signal for autophagy, and treatment with rapamycin induces autophagy in many types of cells and tissues [5, 16]. We investigated whether the inhibition of mTOR via rapamycin treatment in early postnatal life would induce autophagy and enhance cytoplasmic change of mSCs. Rapamycin was injected intraperitoneally beginning on P7 for one week, and the efficacy of rapamycin-induced inhibition of mTOR activity was shown by determining the level of phospho-S6 kinase, a target of mTOR signaling [16] at P14. Rapamycin injection strongly diminished the phosphorylation of S6 kinase but increased LC3B-II levels compared to vehicle-treated animals (Fig. 5A). However, the administration of rapamycin did not affect the expression of MBP and P0 (Fig. 5A), suggesting that the suppression of mTOR activity by rapamycin induced autophagy activation *in vivo*. After the injection of rapamycin or vehicle for one week (at P14), we examined the ultrastructure of the sciatic nerves and measured the abaxonal cytoplasmic area of the mSCs. We found a significant reduction in the abaxonal cytoplasmic area in the mSCs from rapamycin-treated mice compared to vehicle-treated mice (Fig. 5B, C). These findings indicate that the inhibition of the mTOR pathway enhanced cytoplasmic reduction in mSCs, most likely through autophagy activation.

## Discussion

It was known that the abundant abaxonal cytoplasm of mSCs during early postnatal period is gradually reduced from P14 when the maximum rate of myelin deposition reached, and very little cytoplasm remains at adults [2–4]. In this study, we have provided several evidence that autophagy regulates active removal of residual abaxonal cytoplasm of mSCs during myelin maturation. First, we have found that a genetic knockout of an essential autophagy gene, *atg7*, in developing SCs resulted in thickened abaxonal cytoplasm even at adults. This finding was accompanied by the developmental regulation of the canonical autophagic pathway in mSCs, which was demonstrated by morphological and biochemical analysis. In particular, we have found that autophagic machinery such as LC3 and lysosome accumulation was peaked around P21, suggesting a role of autophagy in abaxonal cytoplasm reduction during myelin maturation. Levels of the LC3B-II form, which is localized to autophagosomal membranes, peaked at P21 in mSCs and then diminished abruptly thereafter. Because lysosomal fusion with autophagosomes leads to the destruction of LC3B-II [5], the sudden loss of LC3B staining after P21 may occur due to extensive formation of autophagolysosomes after P21. Indeed, the cytoplasm of mSCs at P28 still shows many lysosomes and LAMP1 staining, which were not found in adults, observed with EM and IF staining. Taken together, these findings suggest that autophagic reduction of abaxonal cytoplasm of mSCs transiently takes place during the maturation period of myelination. However, it should also be noted that autophagy would not take place within the same temporal window in every mSCs because myelination process is temporally heterogeneous and depends on axonal size.

We frequently found abnormally expanded RER and accumulation of electron-dense materials within the lumen of ER in the cytoplasm of the mSCs in the *atg7*-SCKO nerves. Because autophagy inactivation results in cytoplasmic accumulation of misfolded undegraded proteins and/or ubiquitinated proteins [9, 10], the enlarged ER may be resulted from the accumulation of abnormal proteins within ER. In accordance with this, in *atg7*-SCKO nerves, we observed the increase of p62 that plays an important role in inclusion body formation when autophagy is impaired [17, 18]. On the other hand, previous genetic studies using tissue-specific knockout technology have shown cell-autonomous degeneration of autophagy-defective cells, such as neurons and muscles, and the formation of cytoplasmic inclusion is related to the toxicity [9, 10, 19]. However, chronic autophagy deficiency in Schwann cells (up to 9 months) did not result in demyelination phenotypes (data not shown), indicating that the pathologic process might be cell-type specific.

It is possible that the quality control of myelin protein synthesis and cellular organelle development that are achieved by autophagy might be tightly associated with the myelination process or with SC differentiation. However, our results did not support this hypothesis, because the axonal sorting and initial myelination processes took place normally in *atg7* knockout mice. In addition, we found that autophagy appears to be primarily activated when myelination is already in an advanced stage. It was recently reported that SC-specific mTOR null mice exhibited delayed myelination [19], indicating an important role for the mTOR pathway in SC myelination. Although autophagy may be enhanced or abnormally activated in mTOR-null mice, increased autophagy might not be responsible for the defect in myelination in this mutant. In consistency with this finding, it was found that the activation of autophagy with rapamycin during postnatal development did not alter normal myelination *in vivo*. Therefore, these findings suggest a distinct mechanistic regulation of the initiation of myelination and constitutive autophagy. Moreover, we have not found abnormally compacted myelin lamellae in the *atg7*-SCKO nerves at P10 and P60. This finding indicates that cytoplasmic exclusion from closely condensed myelin membranes during the growth of compacted myelin sheath is mainly performed by myelin protein interaction between closely apposed membranes [20], but is not related to autophagic removal of cytoplasm.

Unexpectedly, we have found the hypermyelination of small fibers in the *atg7*–SCKO mutant mice. The increased number of myelinated axons in mutant nerves (2516±234 vs 2349±190, Fig. 4E), even though it was statistically insignificant, may be related to the hypermyelination potential of *atg7*–SCKO mutants. At this moment, we do not know how the loss of *atg7* resulted in hypermyelination in adulthood. Excess residual cytoplasm in the mSCs of the mutant mice may or may not be related to these abnormalities. NCV of *atg7*–SCKO mutant mice was not accelerated compared to the control mice suggesting that the hypermyelination of small fibers does not significantly contribute to NCV (which reflects the value of large myelinated axons). The increased amplitude of CMAP in *atg7*–SCKO mutant mice may be associated with mild increase of the number of myelinating axons. Further studies on the molecular mechanism of abnormal peripheral nerve function in *atg7*-SCKO mice are required.
